# Temporal Evolution of Frontal Plane Center-of-Mass Transfer Asymmetry in Post-Stroke Gait

**DOI:** 10.1109/TNSRE.2025.3559857

**Published:** 2025-04-18

**Authors:** Keng-Hung Shen, Robert Lee, Hao-Yuan Hsiao

**Affiliations:** Department of Kinesiology and Health Education, The University of Texas at Austin, Austin, TX 78712 USA. He is now with the Department of Neuroscience, Washington University in St. Louis, St. Louis, MO 63110 USA; St. David’s Medical Center, Austin, TX 78705 USA; Department of Kinesiology and Health Education, The University of Texas at Austin, Austin, TX 78712 USA

**Keywords:** Gait, stroke, biomechanics, inverted pendulum, balance, foot placement

## Abstract

In typical human gait, the body center-of-mass (CoM) is cyclically transferred towards and supported by each lower extremity. The magnitude of this CoM transfer can be quantified by measuring the minimum mediolateral distance between the CoM and the stance foot during each step. Individuals with hemiparesis due to stroke often show a reduced and more variable CoM transfer magnitude in paretic versus non-paretic steps, which are linked to slower walking speeds and an increased risk of falling. While the commonly observed wider and more variable paretic foot placement at initial contact likely contributes to such frontal plane CoM transfer abnormalities, other factors could continue to adjust the CoM transfer magnitude after initial contact. To understand how the CoM transfer magnitude evolves throughout the transfer process, we derived an inverted-pendulum-based equation that projects the experimentally measured instantaneous mediolateral CoM position and velocity to the CoM transfer magnitude. We first validated our derived equation by demonstrating that CoM transfer magnitude can be predicted by the CoM position and velocity at the end of the double support phase with passive inverted pendulum dynamics. We then investigated how the asymmetry of this projected CoM transfer magnitude between the paretic and non-paretic steps evolves during the transfer process. Our findings revealed that about 54% of the transfer magnitude asymmetry was established at initial contact, predominantly influenced by foot placement, while another 38% was established during the double support phase, partly due to reduced work input from the non-paretic trailing limb. Additionally, the variability in transfer magnitude was augmented during the double support phase in paretic steps. Overall, the present study introduces a physics-based method capable of predicting CoM transfer magnitude in advance of its completion, and our findings highlight the significant contribution of the double support phase, which was previously less explored, to the asymmetries in CoM transfer magnitude and variability. Our results suggest that biomechanical factors during this phase, such as trailing limb work input, could be critical targets for future research and therapeutic interventions.

## I. Introduction

Stroke is a leading cause of disability in the United States [1]. Among stroke survivors, walking ability is a strong predictor of their quality of life and long-term survival rate [2], [3], [4]. Thus, restoring walking ability is one of the most important goals in post-stroke rehabilitation [5]. In typical human gait, precise control of the whole-body center of mass (CoM) in the frontal plane is crucial for both stability and efficient forward progression [6], [7], [8]. Specifically, following each foot strike, the CoM should be transferred laterally towards, but not exceeding, the leading limb. A proper CoM transfer that keeps the CoM close to the stance foot allows adequate time for the swing limb to advance forward [6] and facilitates energy recycling within the elastic tendons of the stance limb [7]. Moreover, maintaining consistent and less variable CoM transfer across steps is associated with a reduced likelihood of falling [9]. Individuals with hemiparesis due to stroke frequently exhibit an asymmetric CoM motion in the frontal plane. This asymmetry includes a greater distance between the CoM and the stance foot [10] and a more variable CoM state (i.e., position and velocity) [11], [12] when the body is transferred towards the paretic (i.e. during paretic steps) versus the non-paretic side (i.e. during non-paretic steps). Such asymmetric CoM transfer motion in the frontal plane is associated with slower walking speeds and a higher risk of falling [10], [13]. Thus, understanding the underlying control mechanisms and restoring a symmetrical frontal plane CoM transfer is an important goal for gait rehabilitation post-stroke [14].

**Fig. 1.**
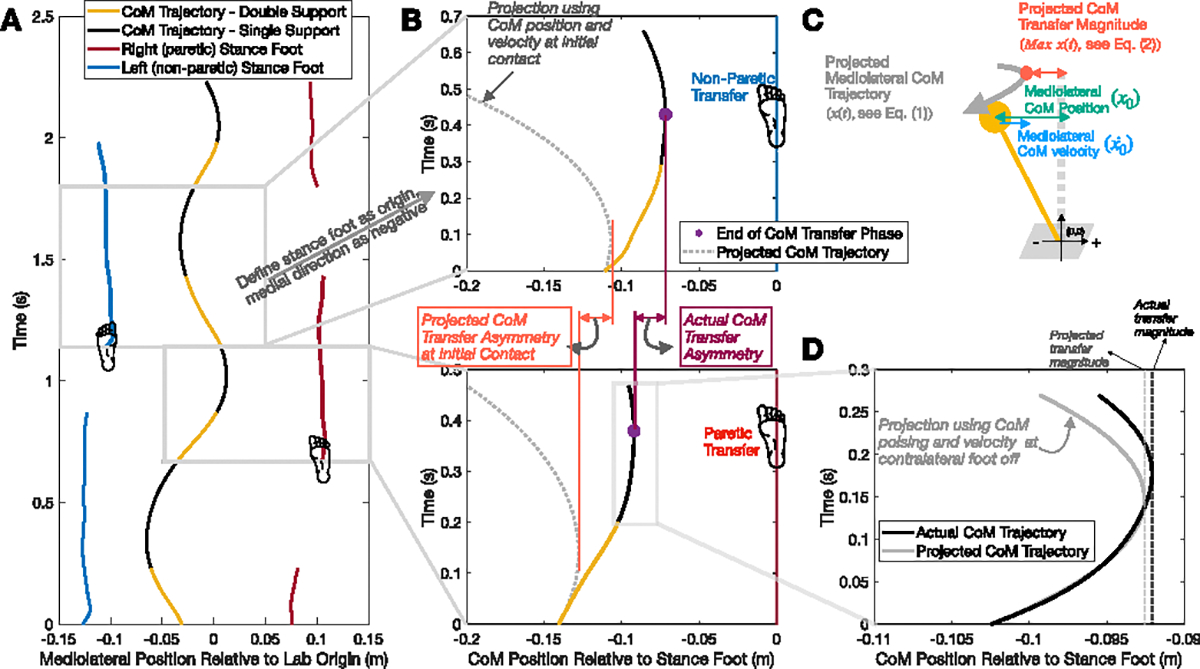
(A) Representative mediolateral trajectories of the center of mass (CoM) and stance feet (right and left) over four steps in the lab coordinate system. (B) Magnified view of two consecutive steps from panel A, presented in the stance foot coordinate system, where the stance foot is defined as the origin and the medial direction is negative. This panel shows the projected mediolateral CoM trajectory (dotted gray lines) based on the CoM state (i.e., position and velocity) at leading limb initial contact, alongside the actual CoM trajectory (solid lines; yellow for the double support phase, black for the single support phase). The calculation of CoM transfer asymmetry is also illustrated. (C) Schematic representation of the projected CoM trajectory *x*(*t*) and CoM transfer magnitude *Max x*(*t*) based on Linear Inverted Pendulum Model (see Eq.(1) and Eq. (2) in the Methods for details). (D) Magnified view of the single stance phase from panel B, depicting the projected CoM trajectory (gray solid line) based on the CoM state at trailing limb foot-off, alongside the actual trajectory (black solid line).

The frontal plane motion in bipedal gait is often modeled as an inverted pendulum [15], [16], [17], with the pendulum point mass representing the whole-body CoM, and the pendulum pivot representing the stance foot (Fig. 1C). In each step, the pendulum CoM is transferred towards the pendulum pivot established at initial contact, reaches the minimum mediolateral distance between the pendulum CoM and the pendulum pivot, and then falls back due to gravity (Fig. 1A). The magnitude of this lateral CoM transfer can be quantified by the minimum mediolateral distance between the pendulum CoM and the pivot point (i.e., the whole body CoM and the stance foot, [10]). Specifically, a smaller distance indicates a larger transfer magnitude. Extensive evidence from the inverted pendulum model and human experiments has emphasized the significant role of foot placement, defined as the mediolateral distance between the CoM and the leading stance foot at initial contact, in regulating frontal plane CoM motion during walking [16], [18], [19], [20], [21], [22]. In individuals post-stroke, studies commonly reported a wider and more variable paretic foot placement compared to the non-paretic limb [12], [23], [24], [25], [26]. While the abnormalities in foot placement likely contribute to a smaller and more variable CoM transfer towards the paretic side, there are other factors that could affect the CoM trajectory based on inverted pendulum dynamics. For example, after foot placement is established at initial contact, the CoM transfer magnitude may be further adjusted by positive work input from the trailing limb. Although adjustments in CoM transfer magnitude after initial contact, together with foot placement, determine the final CoM transfer magnitude, their relative contributions remain unclear.

Integrating diverse biomechanical variables and quantifying their relative contributions to the asymmetries in CoM transfer magnitude and variability presents significant challenges because these variables measure different constructs, belong to different domains, and have different temporal resolutions. For instance, leading limb foot placement and trailing limb work input, two factors influencing CoM transfer dynamics, are measured in different units and occur at different timings, making their relative contributions difficult to assess. To address this challenge, we derived a physics-based equation to link the instantaneous CoM position and velocity to the projected CoM transfer magnitude, defined as the projected nearest distance between the CoM and the stance foot. This equation assumes that the body motion assembles a linear inverted pendulum model (LIPM) given the instantaneous CoM position and velocity without additional external work input afterwards (see Methods. C for details). By converting the instantaneous CoM position and velocity into a single metric (i.e., projected transfer magnitude) expressed in meters, this LIPM-derived equation enabled the comparison of projected CoM transfer magnitude at different time points within a step. With this framework, our overarching goal is to provide a comprehensive understanding of how the projected CoM transfer magnitude and its variability evolves after initial contact. Our central hypothesis is that during paretic steps, the diminished CoM transfer is first established at the paretic initial contact and then compensated by the non-paretic trailing limb, which provides positive work input and attenuates variability with precise step-to-step control.

**Fig. 2.**
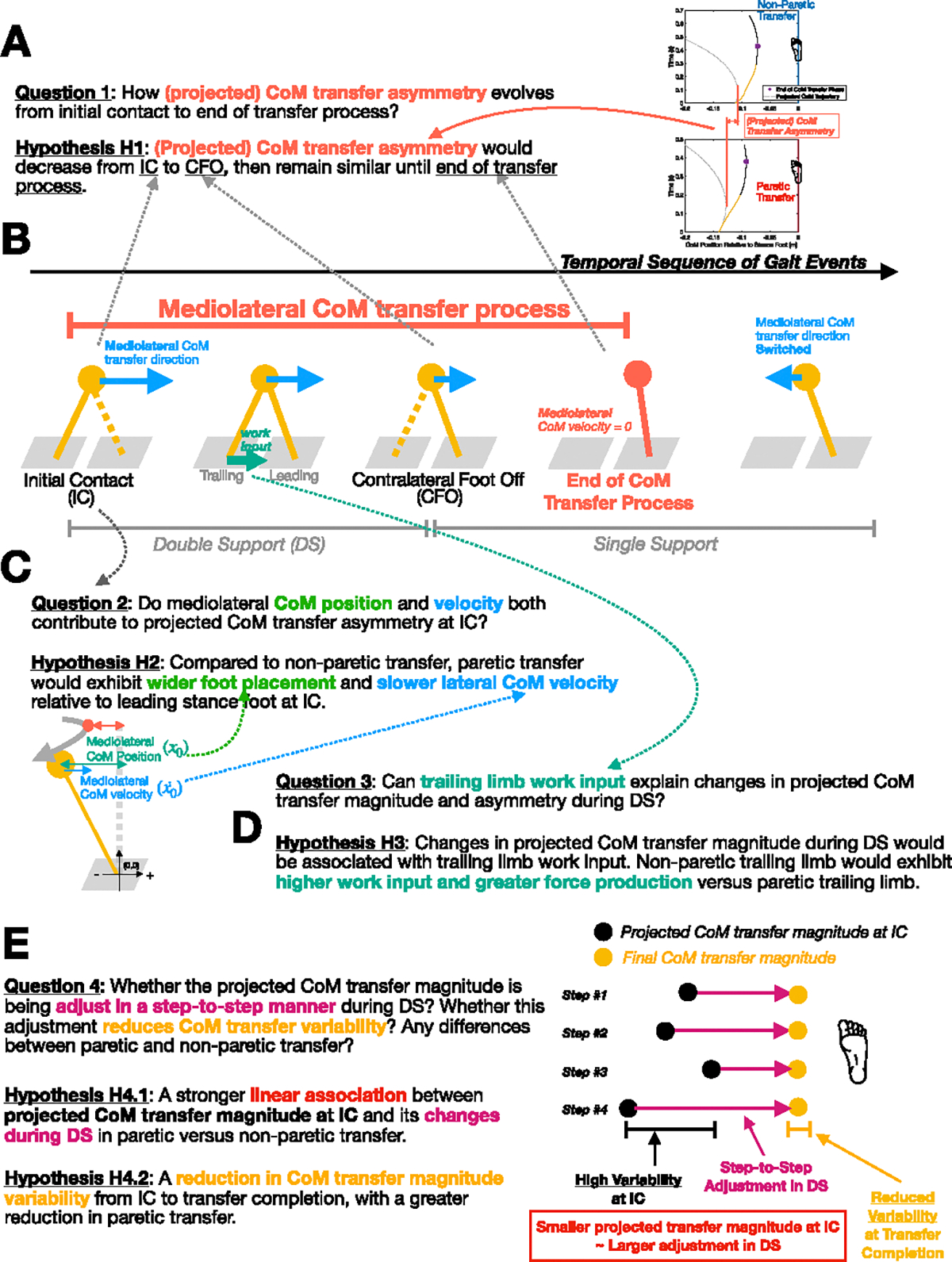
Overview and Schematic Representation of Research Questions and Hypotheses H1 (A), H2 (C), H3 (D), and H4 (E). (B) Illustration of the center-of-mass transfer process and corresponding gait events in the frontal plane. Solid yellow lines indicate the stance limb, while dashed yellow lines represent the limb transitioning into or out of ground contact.

We first validated our LIPM-derived equation by assessing its ability to predict the final CoM transfer magnitude using the CoM position and velocity at trailing limb foot-off (i.e., the end of double support phase; Fig. 1D). This validation approach is based on the expectation that body motion during this phase closely approximates the passive dynamics of a linear inverted pendulum, as external work input is likely negligible after trailing limb foot-off. We then applied the validated equation to investigate the temporal evolution of the CoM transfer magnitude asymmetry between paretic and non-paretic steps throughout the transfer process, defined as the period from initial contact to the moment when the CoM reaches the minimum mediolateral distance to the stance foot (Fig. 2B). Specifically, we compared the CoM transfer magnitude asymmetry across three time points: leading limb initial contact, trailing limb foot off, and at the end of the transfer process, where the asymmetry at the first two time points was calculated from the projected transfer magnitude (Fig. 1B). We hypothesized (H1) that the projected CoM transfer asymmetry would be the highest at initial contact, decrease at trailing limb foot off (end of double support phase), and remain consistent from trailing limb foot off to transfer completion (Fig. 2A). These results would suggest that asymmetry established at initial contact is primarily attenuated during the double support phase. Additionally, we quantified the relative contributions of projected CoM transfer magnitude asymmetry at initial contact and its changes during the double support and early single support phases to the final CoM transfer asymmetry.

Following the analysis of the temporal evolution of CoM transfer magnitude asymmetry, we examined specific biomechanical variables that could influence the projected CoM transfer magnitude during each gait phase. At initial contact, we quantified the relative contribution of the foot placement-related component versus the CoM velocity-related component to the projected CoM transfer magnitude. Because a wider foot placement and a lower CoM velocity both would reduce the projected CoM transfer magnitude based on our LIPM-based equation, we hypothesized (H2) that individuals post-stroke would exhibit wider foot placement and decreased lateral CoM velocity during paretic compared to non-paretic initial contact (Fig. 2C). This result would suggest that both factors contribute to the reduced CoM transfer magnitude toward the paretic side. During the double support phase, we hypothesized (H3) that changes in projected CoM transfer magnitude are associated with trailing limb work input. Additionally, we hypothesized that the non-paretic trailing limb would exhibit higher work input due to greater force generation compared to the paretic trailing limb (Fig. 2D). This result would suggest that the non-paretic trailing limb compensates for the projected CoM transfer magnitude asymmetry established at paretic initial contact by positive work input.

Lastly, we examined the step-to-step adjustment in the projected CoM transfer magnitude during the double support phase and its relationship to the projected CoM transfer magnitude at initial contact. Previous studies in prosthetic foot controller design have demonstrated that using the CoM position and velocity at initial contact to inform trailing limb ankle push-off intensity can enhance frontal plane stability [27], [28]. We aimed to investigate whether this mechanism is also present in post-stroke gait, potentially compensating for the greater variability in paretic foot placement at initial contact [26]. Specifically, we explored whether smaller projected CoM transfer magnitudes at initial contact are associated with larger adjustments during the double support phase. We hypothesized (H4.1) that the projected CoM transfer magnitude at initial contact and its changes during the double support phase would exhibit a stronger negative correlation in paretic steps compared to non-paretic steps (Fig. 2E). This would suggest that the CoM transfer magnitude is more tightly regulated after initial contact in paretic steps, likely achieved through the work input of the non-paretic trailing limb. Furthermore, we examined whether the aforementioned control mechanism is reflected in the variability of the CoM transfer magnitude. We hypothesized (H4.2) a reduction in CoM transfer magnitude variability from initial contact to CoM transfer completion in both paretic and non-paretic steps due to step-to-step adjustment, with a greater reduction in paretic steps (Fig. 2E).

In the present study, we characterized the temporal evolution of frontal plane CoM transfer dynamics in individuals post-stroke. By converting experimentally acquired CoM position and velocity at each critical time point to projected CoM transfer magnitude using inverted pendulum dynamics, we identified when and how the asymmetries in transfer magnitude and variability are established. This approach narrowed the temporal window for identifying biomechanical factors contributing to CoM transfer asymmetry. Our findings revealed crucial biomechanical targets and their relative contributions underlying frontal plane CoM transfer asymmetry, which could inform the development of rehabilitation interventions to improve post-stroke ambulatory function.

## II. Materials and Methods

### A. Participants

Fourteen individuals post-stroke participated in the present study. The inclusion criteria for study participants are: (1) Residual hemiparesis due to chronic stroke greater than 6 months prior to the study, (2) Able to walk 10 meters with or without a walking aid, and (3) Able to stand unsupported for 5 minutes. Exclusion criteria include (1) Medical condition beyond the effects of the stroke that preclude participation in regular exercise, (2) Bilateral strokes or a previous stroke in the contralateral hemisphere, (3) Cerebellar stroke. The study was approved by the University of Texas at Austin Institutional Review Board on 4/25/2022 (IRB ID: STUDY00002635). The participants were recruited as part of a clinical trial: Treadmill Oscillation Walking to Improve Weight Transfer During Gait Following Stroke (ClinicalTrials.gov ID: NCT05541705). All participants provided written informed consent to participate.

As our analysis employed inverted pendulum dynamics, which could be influenced by handrail use, one participant who required handrail support for every step was excluded. Additionally, since the study focused on the mechanisms underlying reduced CoM transfer toward the paretic limb in post-stroke gait, two participants whose kinematic data did not show reduced CoM transfer during paretic compared to non-paretic steps were excluded from most analyses. These two participants were retained for validating our LIPM-based CoM transfer magnitude prediction (Result. A), as inverted pendulum dynamics was expected to be applicable regardless of the presence of transfer asymmetry. Their temporal evolution patterns of projected CoM transfer magnitude are presented in Figure 4A to provide a comprehensive representation of the participant cohort. Consequently, eleven participants were included in analyses in Result. B-E (Table I).

### B. Experimental Protocol

#### 1) Biomechanical Data Collection:

We first determined the participants’ comfortable walking speed on an instrumented treadmill (Motek Inc., US). The treadmill speed was adjusted in gradual increments of 0.05 m/s until the participants identified a speed that felt natural and comfortable, with only occasional need for handrail support. Once identified, participants were given at least one minute to familiarize themselves with walking at this speed on the treadmill.

Next, 72 reflective markers were attached to the participant’s each segment, including the head, pelvis, trunk, bilateral humerus, forearm, thigh, shank, and feet. For each segment at the upper and lower extremities, four markers were attached to the medial and lateral bony landmarks at the segment’s distal and proximal ends, and a cluster of three to four markers was attached to the lateral side of the segment. Pelvis was tracked by four markers placed at the anterior and posterior iliac spines, and the trunk was tracked by six markers placed at sternum, clavicle, 7^th^ cervical spine, 10^th^ thoracic spine, and bilateral acromion. Head was tracked by four markers placed at bilateral front and back of the head.

After attaching the markers, the participant walked on the instrumented treadmill at their comfortable speed, during which a one-minute walking trial was recorded. The participant wore a safety harness, which did not provide body weight support, to ensure safety during the treadmill session. Kinematic data were captured using a 10-camera Vicon motion capture system (Vicon, US) with a sampling rate of 100 Hz. Ground reaction forces and center-of-pressure (CoP) data were recorded by an instrumented split-belt treadmill sampling at 1000 Hz. Both kinematic and kinetic data were filtered offline using a fourth-order Butterworth low-pass filter with cutoff frequencies of 6 Hz for kinematic data and 15 Hz for kinetic data.

#### 2) Clinical Measures:

On a different day within 7 days of the biomechanical data collection, participants completed a battery of clinical measures, including Berg Balance Score [29] and 10-Meter Walk Test at self-selected speed [30], evaluated by a licensed physical therapist. During the evaluation, the participants were allowed to use the external assistance that they typically use in daily ambulation (walkers, canes, or trekking poles; Table I).

### C. CoM Transfer Magnitude Projection With the Linear Inverted Pendulum Model

Linear inverted pendulum model (LIPM), a two-dimensional inverted pendulum variant that has been widely used for understanding balance control in humanoid bipedal walking [16], [21], [31], [32], was employed to derive the equation for converting instantaneous CoM position and velocity to the projected mediolateral CoM transfer magnitude. Here, we first detailed the derivation of the equation and then specified the experimental data input used.

#### 1) Equation Derivation for LIPM-Based CoM Transfer Magnitude Projection:

In the LIPM, the pendulum CoM is a point mass, and the model assumes a constant height of the pendulum CoM [31]. The horizontal trajectory of the LIPM is described by the following equation, where $x_{0}$ is the mediolateral position of the pendulum CoM relative to the pendulum pivot (in meters), and ω is the eigenfrequency of the pendulum, calculated as $\sqrt{\frac{g}{l}}$ with $g=9.81\;m/s^{2}$ and l being the instantaneous pendulum CoM height:

(1) $$x\left(t\right)=x_{0}\;cosh(\omega t)+\frac{\dot{x_{0}}}{\omega }\;sinh(\omega t)$$

As illustrated in Fig. 1C, the pendulum pivot point is set at (0, 0), and the mediolateral direction pointing from the pivot to the CoM is defined as negative, making $x_{0}$ a negative scalar. During CoM transfer, as the pendulum point mass moves towards its pivot point, $\dot{x_{0}}$ (the time derivative of $x_{0}$, in m/s) is a positive scalar during this interval.

We defined CoM transfer magnitude as the minimal distance between the pendulum point mass and the pivot point throughout the stance phase. To acquire this magnitude, we differentiated (1) with respect to time and solved for when the rate of change of the trajectory equals zero:

$$\begin{array}{l} \dot{x}\left(t\right)=\frac{d}{dt}\left(x_{0}\;\cosh\left(\omega t\right)+\frac{{\dot{x}}_{0}}{\omega }\;\sinh\left(\omega t\right)\right)\\ \;=x_{0}\sinh(\omega t)+\frac{{\dot{x}}_{0}}{\omega }\;\cosh(\omega t) \end{array}$$

Setting $\dot{x}(t)=0$ provides:

$$\omega t={tan}^{-1}\left(\frac{\dot{x_{0}}}{\omega x_{0}}\right)$$

This solution is valid if $\left|\frac{\dot{x_{0}}}{\omega x_{0}}\right|\leq 1$, ensuring the calculation remains within real numbers. $\frac{\dot{x_{0}}}{\omega x_{0}}>1$ indicates that the LIPM would fall laterally over the pivot, and no minimum distance could be defined. Substituting ωt back into (1) results in the following equation:

(2) $$Max\;x(t)=x_{0}\;cosh\left({tan}^{-1}\left(\frac{\dot{x_{0}}}{\omega x_{0}}\right)\right)\;\;+\frac{\dot{x_{0}}}{\omega }\;sinh\left({tan}^{-1}\left(\frac{\dot{x_{0}}}{\omega x_{0}}\right)\right)$$

#### 2) Decomposing the LIPM-Based CoM Transfer Magnitude Projection Equation:

To assess the relative contributions of foot placement (FP) and CoM velocity (CoM_v) on projected CoM transfer magnitude at IC, we further decomposed (2) into two components, one related to FP and the other to CoM_v, each associated with their respective coefficients:

$$\mathrm{FP-related}\;\mathrm{Component}\;=x_{0}\;cosh\left({tan}^{-1}\left(\frac{\dot{x_{0}}}{\omega x_{0}}\right)\right)$$

$$FP\;\mathrm{Coefficient}=cosh\left({tan}^{-1}\left(\frac{\dot{x_{0}}}{\omega x_{0}}\right)\right)$$

$$\mathrm{CoM\_v-related}\;\mathrm{Component}=\frac{\dot{x_{0}}}{\omega }\;sinh\left({tan}^{-1}\left(\frac{\dot{x_{0}}}{\omega x_{0}}\right)\right)$$

$$\mathrm{CoM\_v}\;\mathrm{Coefficient}=\frac{1}{\omega }\;sinh\left({tan}^{-1}\left(\frac{\dot{x_{0}}}{\omega x_{0}}\right)\right)$$

#### 3) Experimental Data Used in the Equation:

By modeling the human as an linear inverted pendulum in the frontal plane, where the pendulum CoM is the whole-body CoM, and the pendulum pivot point is the ground projection of the leading stance foot CoM, we calculated the projected CoM transfer magnitude by inputting the horizontal position ($x_{0}$), velocity ($\dot{x_{0}}$), and height (l) of the CoM at a specific instant into (2). The result is a negative scalar because the CoM is medially located relative to the stance foot in a step.

Across our participants, the maximum CoM height changes during the CoM transfer phase were 0.72 ± 0.26% and 1.27±0.41% body height for transfers towards the paretic and non-paretic sides, respectively. These small changes suggest that the assumption of constant CoM height per the LIPM definition is valid.

### D. Outcome Measures Calculation

Using Visual 3D software (C-motion, US), a 12-segment model (head, trunk, pelvis, bilateral humeri, forearms, thighs, shanks, and feet) was constructed, and the CoM for the model and each foot segment was calculated. We manually labeled and excluded from the analysis any steps that involved handrail use, foot crossover to the contralateral belt, or toe drag during the paretic swing phase by visually inspecting each walking trial. All other calculations were performed using custom algorithms in MATLAB (MathWorks, US).

To investigate the temporal evolution of CoM transfer asymmetry in post-stroke gait, three gait events were identified: (a) initial contact (IC), (b) contralateral foot off (CFO), and (c) CoM transfer completion. Initial contact and contralateral foot off instants were defined using kinetic data. Completion of CoM transfer was determined at the moment when the minimum mediolateral distance between the CoM and the ground projection of the stance foot CoM occurred during the single stance phase.

At IC and CFO, projected CoM transfer magnitude was calculated using (2). Final CoM transfer magnitude at transfer completion was defined as the minimum distance between the CoM and the stance foot during the single stance phase. We calculated the asymmetry at the three gait event instants by averaging the CoM transfer magnitude within each side for each participant and subtracting the value in paretic step from the non-paretic step (Fig. 1B). Based on our definition of the mediolateral direction, all CoM transfer magnitudes were negative numbers, with their absolute values representing the distance. Therefore, a positive asymmetry value indicates a greater CoM transfer toward the non-paretic side.

We further analyzed the relative contributions of projected CoM transfer asymmetry at different gait phases to final asymmetry. Since final CoM transfer asymmetry is the sum of the projected CoM transfer asymmetry at IC and its changes during the double support (from IC to CFO) and early single support (from CFO to CoM transfer completion) phases, their contributions were quantified as follows:

$$IC\;Contribution\;=\left(\frac{\mathrm{Projected}\;\mathrm{Asymmetry}\;\mathrm{at}\;\mathrm{IC}}{\mathrm{Final}\;\mathrm{CoM}\;\mathrm{Transfer}\;\mathrm{Asymmetry}}\right)\times 100\%$$

$$Double\;Support\;(or\;Early\;Single\;Support)\;Contribution\;=\left(\frac{\mathrm{Changes}\;\mathrm{in}\;\mathrm{Projected}\;\mathrm{Asymmetry}}{\mathrm{Final}\;\mathrm{CoM}\;\mathrm{Transfer}\;\mathrm{Asymmetry}}\right)\times 100\%$$

To quantify the predictive accuracy of final CoM transfer magnitude using (2) with CoM position and velocity at CFO, we calculated the mean absolute error (MAE) between the predicted and final CoM transfer magnitudes across steps for each side of each participant.

As changes in projected CoM transfer magnitude during the double support phase likely reflected work input from the trailing limb, we quantified it using kinetic data. Instantaneous trailing limb power was calculated as the product of mediolateral ground reaction force and CoM velocity. Total work was obtained by integrating instantaneous trailing limb power over the double support phase. Additionally, average power, mediolateral ground reaction force, and CoM velocity during the double support phase were analyzed to explore the components contributing to work input.

To assess the step-by-step control of CoM transfer magnitude, Pearson’s correlation coefficient between the projected CoM transfer magnitude at initial contact and its change during the double support phase was calculated across steps for each side of each participant.

To measure the CoM transfer variability, the standard deviation of the final CoM transfer magnitude and its projected value at IC across steps was calculated for each side of each participant

### E. Statistical Analysis

To test H1 (see Fig. 2 for hypotheses overview), one-way repeated measures ANOVA was performed with the dependent variable being CoM transfer asymmetry, tested across three gait events (IC, CFO, CoM transfer completion) as within-subject factors. Post-hoc analysis with Tukey’s correction was conducted following significant main effect. Additionally, a one-sample, one-tailed t-test was employed to determine if the relative contributions of each gait phase (IC, changes in double support and early single support phases) were significantly greater than zero.

Linear mixed-effects models were used to compare CoM transfer in paretic versus non-paretic step for the following dependent variables, listed according to their corresponding subsection in the Result:

Result. C (H2): FP and CoM velocity at IC.

Result. D (H3): Trailing limb work input, average power, mediolateral ground reaction force, and CoM velocity during double support phase.

Result. E (H4.1): Pearson’s correlation coefficient between the projected CoM transfer magnitude at initial contact and its change during the double support phase.

For each dependent variable, a separate linear mixed-effects model was built. Each model included CoM transfer direction (paretic versus non-paretic) as a fixed effect and subject ID as a random intercept:

DependentVariable~TransferDirection+(1|subjectID).

The significance of the fixed effect (CoM transfer direction) was assessed using a Wald F-test with Kenward-Roger degrees of freedom adjustment.

To test H4.2, another linear mixed-effects model was used to compare CoM transfer variability at different time points and between paretic and non-paretic steps. The model included CoM transfer direction (paretic versus non-paretic) and the timing of variability measurement (at initial contact versus transfer completion) as fixed effects, along with their interaction. Subject ID was included as a random intercept. The model can be expressed as:

$$\begin{array}{l} \mathrm{CoM}\;\mathrm{Transfer}\;\mathrm{Variability}\;\sim \;\mathrm{Transfer}\;\mathrm{Direction}\;+\;\mathrm{Time}\;\mathrm{Point}\\ \;+\;(\mathrm{Transfer}\;\mathrm{Direction}\;\times \;\mathrm{Time}\;\mathrm{Point})\\ \;+\;(1|\;\mathrm{Subject}\;\mathrm{ID}) \end{array}$$

The significance of the fixed effects and their interaction were determined using the Wald F-test with Kenward-Roger degree of freedom adjustment. Planned post-hoc comparisons were conducted to compare CoM transfer variability between limbs at each time point and across time points within each limb transfer direction. Accordingly, four pairwise comparisons were conducted with Tukey’s correction.

Simple linear regression was used to quantify the variance of changes in projected CoM transfer magnitude accounted for by the kinetically derived work input during the double support phase (H3).

All statistical analyses were performed using R [33]. Normality of residuals for each model was inspected using histograms and Q-Q plots. The p-values were adjusted using the Benjamini-Hochberg procedure [34] to control the false discovery rate at 5%.

## III. Result

In Table. II, we list each hypothesis alongside the corresponding results, indicating whether they are supported or not by our findings.

### A. LIPM-Based CoM Transfer Magnitude Prediction Validation

**Fig. 3.**
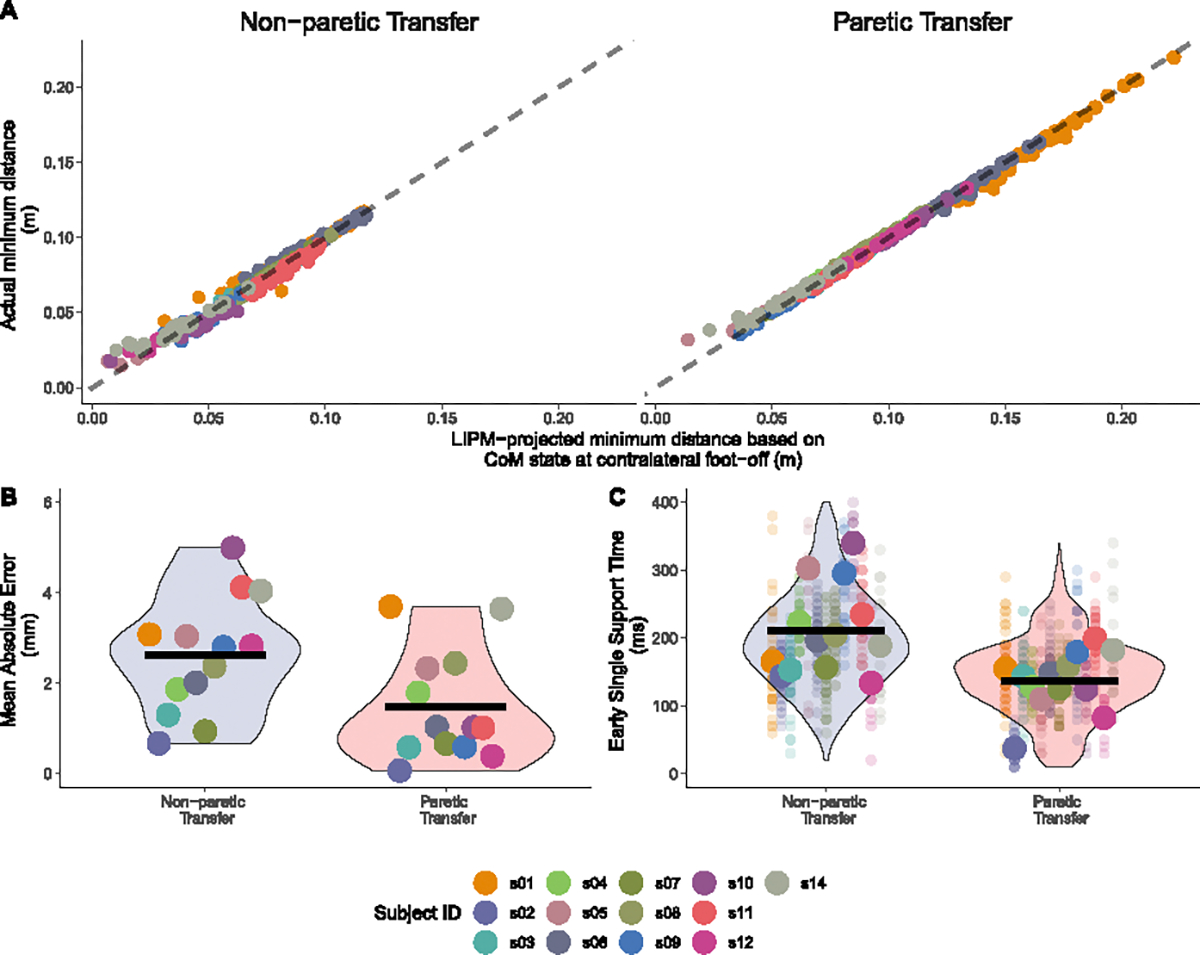
Linear Inverted Pendulum Model (LIPM)-based CoM transfer prediction validation. (A) The association between LIPM-projected CoM transfer magnitudes at contralateral foot off and final magnitudes for both paretic (right) and non-paretic (left) steps. Dotted gray lines represent the identity line (*y* = *x*). Each point represents a step from a participant. Different colors code data from different participants. The two participants (s7 and s11) who did not show a smaller CoM transfer magnitude towards the paretic versus non-paretic side are included in this validation analysis. (B) Mean absolute error in CoM transfer predictions, with each point representing a result from each participant. Violin plots demonstrate the distribution across participants, and horizontal black lines indicate the grand means. (C) Early single support time (from contralateral foot off to CoM transfer completion) for both paretic and non-paretic transfers. Each large circle represents averaged results from each participant, while transparent points represent individual steps. Violin plots show the distribution across steps, and horizontal black lines mark the grand means. Different colors code data from different participants.

We first validated the predictive accuracy of LIPM-based CoM transfer magnitude estimation at CFO for predicting the final CoM transfer magnitude. Data points of predicted versus actual CoM transfer magnitudes closely aligned with the identity line (y = x), qualitatively indicating a strong predictive accuracy (Fig. 3A). The mean and the standard deviation of the MAE of the predictions were 1.48 ± 1.20 and 2.62 ± 1.28 (mm) for the paretic and non-paretic transfers, respectively (Fig. 3B). These prediction errors fall within the typical range of marker-based motion capture systems (1–5 mm) [35], [36], supporting model validity. Additionally, the early single support time averaged 136 ± 44 and 211 ± 66 (ms) for the paretic and non-paretic transfers, respectively (Fig. 3C).

### B. Temporal Evolution of CoM Transfer Asymmetry (H1)

**Fig. 4.**
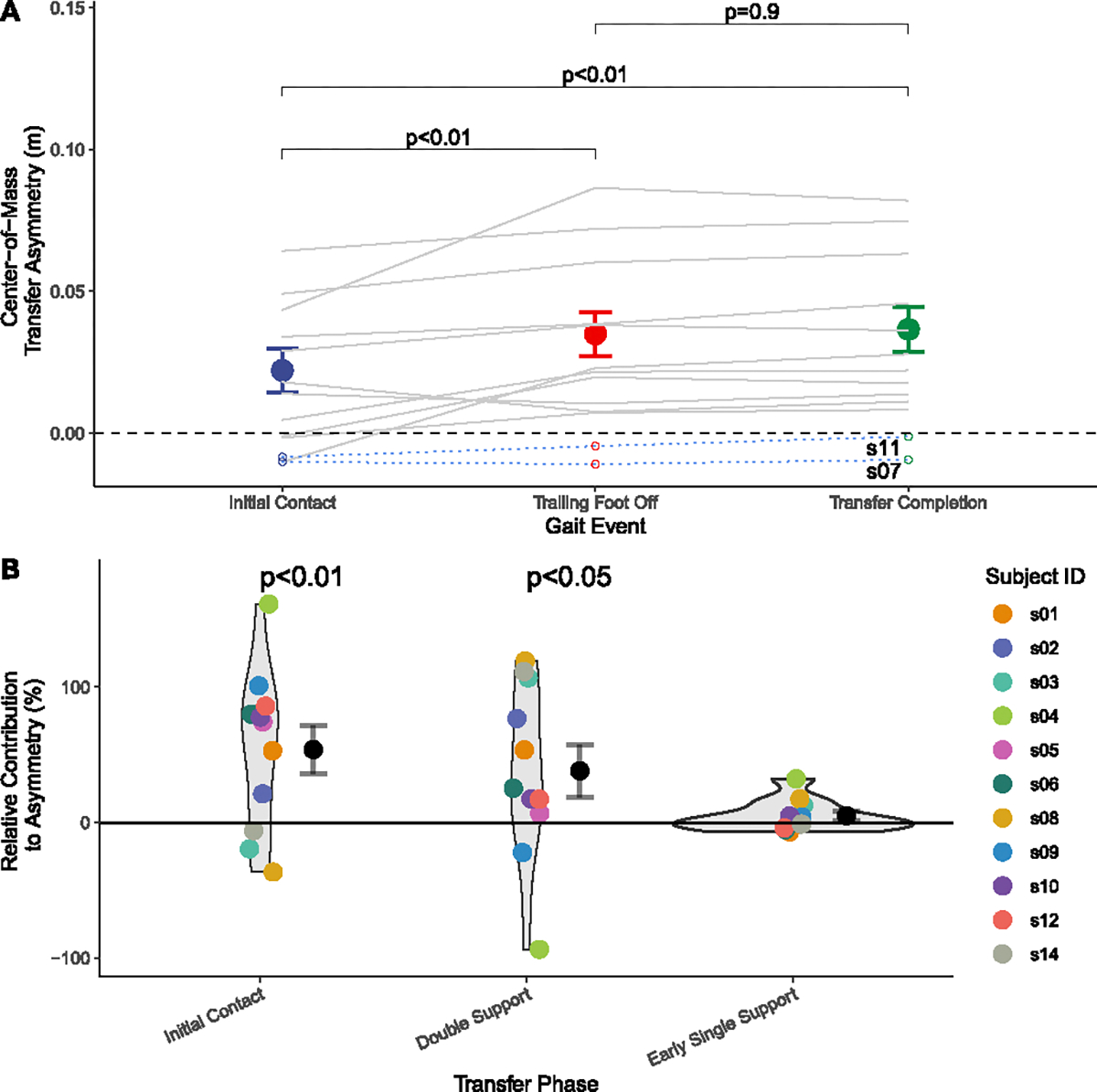
Temporal evolution of CoM transfer asymmetry across different gait events. (A) Comparison of CoM transfer asymmetry across gait events. Data from the same participant are linked with gray lines. Data from the two participants (s7 and s11) who did not show a smaller CoM transfer magnitude towards the paretic versus non-paretic side are shown here but were not included in the statistical tests. (B) The relative contributions of each transfer phase to final CoM transfer asymmetry, with statistical significance against zero noted. Different colors code data from different participants. Black points and error bars represent mean±standard error.

A one-way repeated ANOVA showed a significant main effect of gait event on CoM transfer asymmetry (*p* < 0.01). Post-hoc analysis showed that the projected CoM transfer asymmetry at IC was lower than at CFO (*p* < 0.01) and at CoM transfer completion (*p* < 0.01; Fig. 4A), which partially contradicted our hypothesis H1 (see Table. II).

Additionally, the relative contribution of each gait phase (IC, changes in double support and early single support phases) to final CoM transfer asymmetry was, on average, greater than zero (all *p* < 0.01; Fig. 4B). The projected CoM transfer asymmetry at IC contributed to 53.9% of final CoM transfer asymmetry. Change during the double support phase accounted for 38.1%, while the change during the early single support phase accounted for 4.9% of final CoM transfer asymmetry.

### C. Components Contribute to CoM Transfer Asymmetry at Initial Contact (H2)

**Fig. 5.**
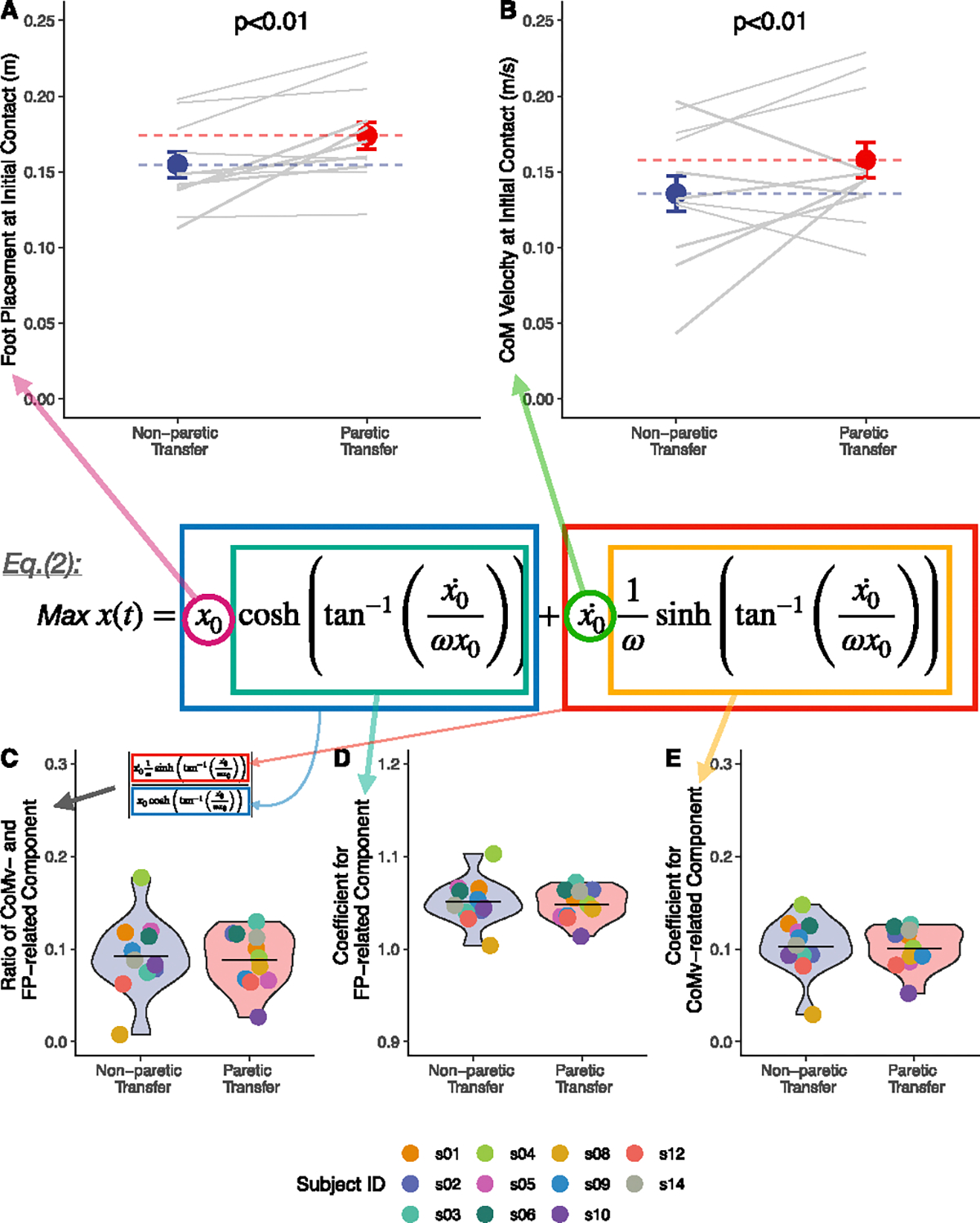
Components contributing to CoM transfer asymmetry at initial contact. (A-B) Comparing foot placement (FP) and center-of-mass (CoM) velocity at initial contact between paretic versus non-paretic steps. Data from each participant is linked with a gray line. (C-E) The ratio between and coefficients for FP and CoM_v related components that contributed to projected CoM transfer magnitude at initial contact. Different colors code data from different participants, with violin plot showing the distribution across participants and horizontal black lines indicating the grand means.

The FP and CoM velocity at IC were both greater during paretic versus non-paretic steps (*p* < 0.01; Fig. 5A–B), which partially supported our hypothesis H2 (see Table. II).

Additionally, we investigated the ratio of the magnitude between the CoM_v-related and FP-related components ($\frac{|\mathrm{CoM\_v-related}\;\mathrm{Component}|}{\left|FP-related\;Component\right|}$; see Methods. C.2 for details) in the LIPM-based equation to assess the compensatory effect of CoM velocity on FP at IC. The ratio was approximately 0.09 for both paretic (0.09 ± 0.04) and non-paretic (0.09 ± 0.03) transfers (Fig. 5C). The ratio predominantly arose from the coefficients of each component, which exhibit a tenfold difference in scale between the FP and CoM_v components. Specifically, for paretic transfers, the FP coefficient was 1.05 ± 0.02 and the CoM_v coefficient was 0.10 ± 0.02; for non-paretic transfers, the coefficients were 1.05 ± 0.02 for FP and 0.10 ± 0.03 for CoM_v, respectively (Fig. 5D–E).

### D. Kinetically Derived Work Input (H3)

**Fig. 6.**
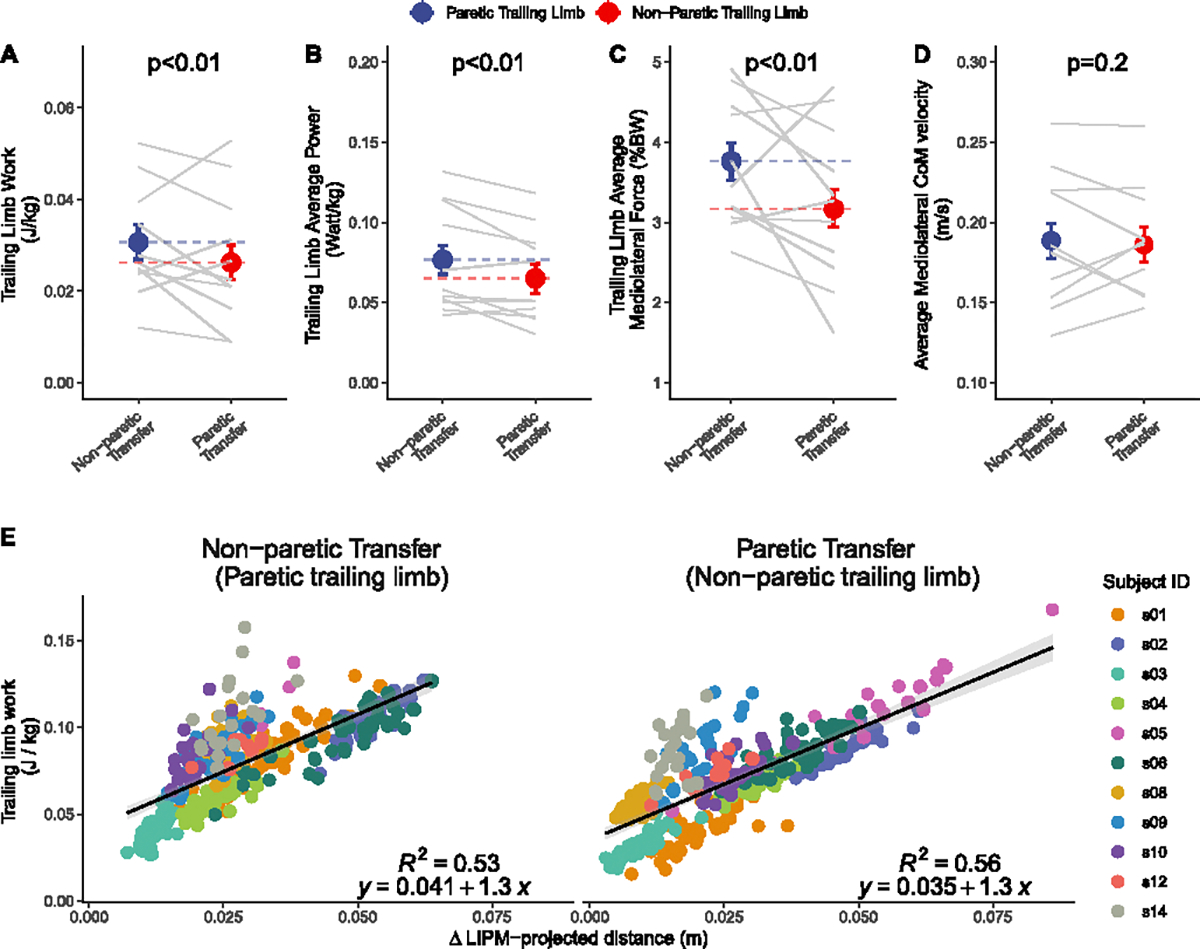
Kinetically derived work input during the double support phase. (A-D) Comparisons in work input, average power, average mediolateral ground reaction force, and average center-of-mass velocity between paretic and non-paretic transfers. Data from each participant is linked with a gray line. (E) The association between the trailing limb mediolateral work input and changes in projected CoM transfer magnitude during the double support phase. Regression results are shown by solid black lines. Each point represents a step from a participant. Different colors code data from different participants. *LIPM: Linear Inverted Pendulum Model.*

Over 50% of the variance in the change in projected CoM transfer magnitude during the double support phase was explained by trailing limb total work input (Paretic: *R*^2^ = 0.56; Paretic: *R*^2^ = 0.53), which supported our hypothesis H3 (see Table. II). The unstandardized slopes were 1.3 for both transfer directions (Fig. 6E).

During the double support phase, the trailing limb work input, average power, and average mediolateral ground reaction force were higher when the body was transferred towards the non-paretic versus the paretic side (*p* < 0.01 for all three metrics; Fig. 6A–C), which contradicted our hypothesis H3 (see Table. II). The average CoM velocity during the double support phase showed no significant difference between transfers towards the paretic and non-paretic limbs (*p* = 0.2; Fig. 6D).

### E. Step-by-Step Control of Work Input During Double Support Phase (H4)

**Fig. 7.**
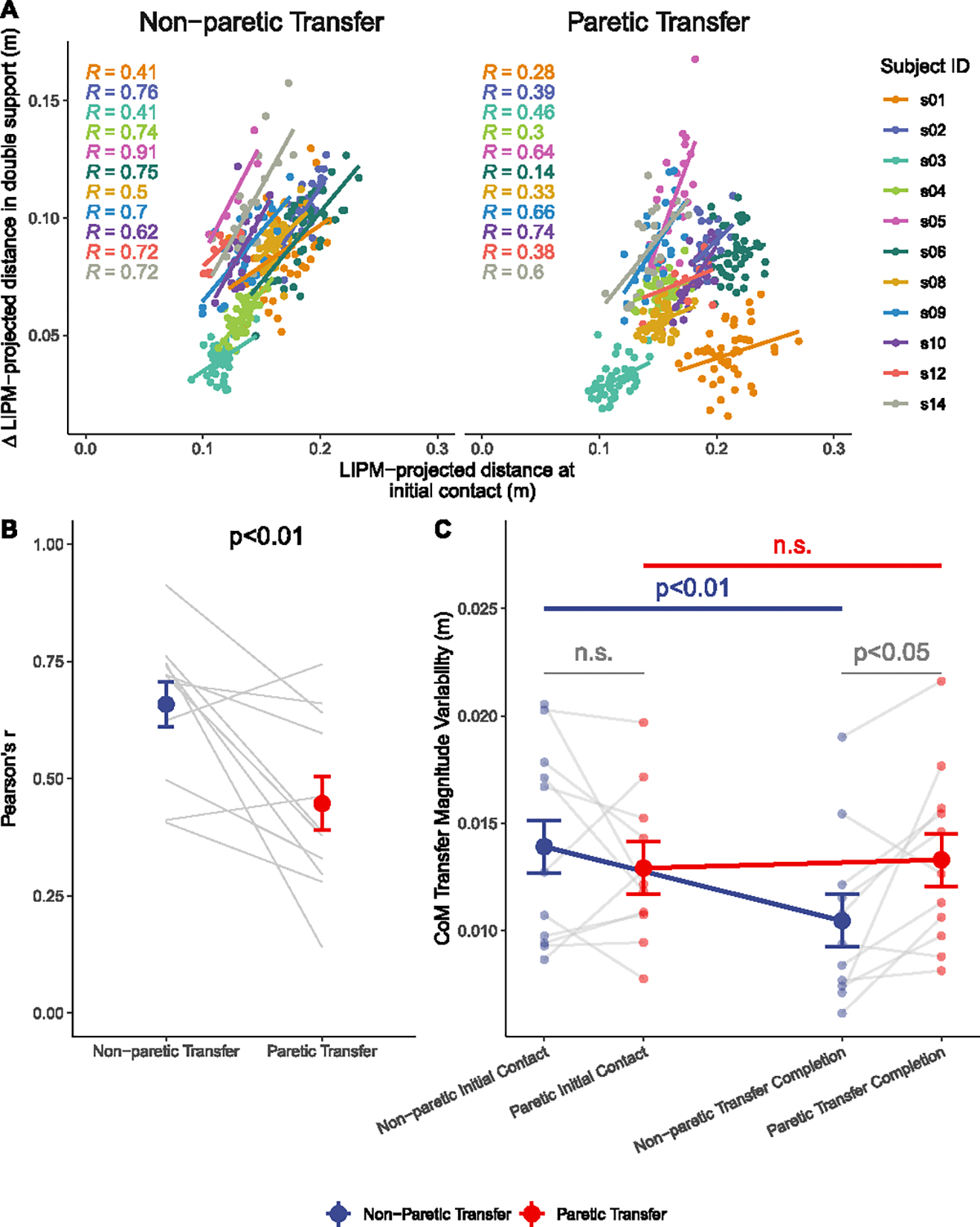
Step-by-step adjustment of CoM transfer magnitude during the double support phase. (A) The association between projected CoM transfer magnitudes at initial contact and its changes during the double support phase for both paretic and non-paretic steps. Different color indicating data from each participant. The best fit line for each participant is indicated by a solid line, with each point represents a step from a participant. (B) Comparison of Pearson’s correlation coefficient (r) between paretic and non-paretic transfers. Data from each participant is linked with a gray line. (C) Comparisons of CoM transfer variability between paretic and non-paretic transfers at initial contact and transfer completion, and between initial contact and transfer completion for each transfer direction. *LIPM: Linear Inverted Pendulum Model.*

The strength of the relationship between the projected CoM transfer magnitude at IC and its changes during the double support phase, quantified by Pearson’s correlation coefficient (Fig. 7A), was weaker for the paretic steps compared to the non-paretic steps (*p* < 0.01; Fig. 7B), which contradicted our hypothesis H4.1 (see Table. II).

When comparing the transfer variability at different gait events and across limbs, a significant main effect of gait event (*p* < 0.05) and its interaction with transfer direction (*p* < 0.05) was found, while the effect of transfer direction alone was not significant (*p* = 0.19). Planned post-hoc comparisons showed that the variability was significantly reduced from IC to transfer completion in non-paretic steps (*p* < 0.01), whereas no difference in transfer variability between IC and transfer completion was found in paretic steps (*p* = 0.99; Fig. 7C), which partially contradicted our hypothesis H4.2 (see Table. II). Additionally, the variability was greater when the body was transferred towards the paretic versus the non-paretic side at transfer completion (*p* < 0.05), but no between-limb differences was found at IC (*p* = 0.75; Fig. 7C).

## IV. Discussion

By linking the instantaneous CoM position and velocity to the projected CoM transfer magnitude through the LIPM dynamics, we were able to compare the projected CoM transfer magnitude across different gait events. Our findings reveal that reduced work input from the non-paretic trailing limb during the double support phase, along with the smaller projected CoM transfer magnitude at IC primarily influenced by wider paretic foot placement, are major factors leading to reduced CoM transfer magnitude towards the paretic side. Additionally, adjustments in the projected CoM transfer magnitude during the double support phase contributed to increased variability in paretic steps. Furthermore, our results demonstrate that the CoM transfer magnitude can be predicted from the CoM state at CFO using passive inverted pendulum dynamics, indicating minimal work input from CFO to transfer completion. Collectively, these findings provide a comprehensive understanding of the critical phases and biomechanical factors contributing to the asymmetries in CoM transfer magnitude and variability in individuals post-stroke, and highlight the previously less explored double support phase as an important target for future therapeutic interventions.

The core of our approach involves the development of LIPM-based CoM transfer magnitude projection, which may be confused with the concept of Margin of Stability (MOS), another LIPM-derived balance measure. The key distinction is that while MOS describes CoM mechanics in terms of mechanical impulse, our approach provides a more comprehensive representation of the mechanical energy state. The MOS, quantified as $x_{0}+\frac{{\dot{x}}_{0}}{\omega }$, represents the instantaneous impulse required to destabilize the CoM and cause a lateral fall [16]. However, identical MOS values can result from different CoM velocities, and the same impulse applied to the CoM at varying velocities produces distinct changes in CoM kinetic energy. Consider an inverted pendulum with some initial velocity, if no additional work is introduced, the passive motion of pendulum CoM towards its pivot reflects the kinetic-to-potential energy transfer process. When all kinetic energy is fully transformed into potential energy, the CoM reaches its closest point to the pivot. This moment corresponds to transfer completion in our study, and the transfer magnitude can be interpreted as a measure of the potential energy magnitude. Thus, our LIPM-based projected CoM transfer magnitude represents the instantaneous mechanical energy state of the CoM, projected to the moment when all energy is converted into potential energy. Accordingly, any change in this estimate would indicate external work input. Indeed, during double support, we observed that changes in projected CoM transfer magnitude correlate with trailing limb work input. Additionally, while minimum MOS typically occurs mid-double support in healthy gait [32], our projected CoM transfer magnitude continues to increase until the end of the double support phase when the trailing limb ceases to add positive work. Lastly, our approach accurately predicts the CoM transfer magnitude based on the CoM state at CFO, indicating minimal work input between CFO and transfer completion in individuals post-stroke. By focusing on mechanical energy state, our method complements MOS by capturing gait mechanics not reflected in impulse-based measurements and providing predictive power over kinematic outcomes across temporally distant events during which no energy input occurs.

Our results demonstrated that the CoM position and velocity at CFO can predict the CoM transfer magnitude with a mean absolute error of approximately 2 mm. This prediction also provides an average lead time of over 100 ms before the transfer is completed. This highlights the capability of our equations to deliver precise kinematic predictions. Recent research have suggested that effective balance restoration with an exoskeleton necessitates reaction speeds exceeding physiological limits [37]. Our equation could provide critical applications in wearable robotics by enabling the prediction of future CoM motion, thereby facilitating timely adjustments. Additionally, the application of our LIPM-based equation for frontal plane CoM motion prediction may potentially be extended to healthy controls and other clinical populations, though validation through future research is warranted. The close approximation of CoM motion by a passive inverted pendulum in our result also suggests minimal work input during the early single stance phase. Previous research has observed significant changes in whole-body angular momentum during the paretic single stance phase in individuals post-stroke [38], [39], and this enlarged rotational motion is associated with poor balance control. Modeling the human body as a flywheel-actuated inverted pendulum, changes in whole-body angular momentum can introduce additional work to the pendulum CoM [21], [40], [41], potentially explaining the correlation between increased angular momentum and reduced balance scores as the added work could disrupt balance if excessive. However, our results of minimal work input during early single stance suggest that the previously identified fluctuation in angular momentum may not substantially affect CoM motion during the paretic single stance phase. Therefore, instead of affecting linear CoM motion, the significant change in angular momentum likely represents a distinct aspect of balance. This supports the notion that no single measure, such as MOS or angular momentum, should be exclusively relied upon, as each captures different facets of dynamic balance [42].

### A. Components Contribute to CoM Transfer Asymmetry at Initial Contact (H2)

Our results indicate that wider foot placement is the primary factor contributing to projected CoM transfer magnitude asymmetry at IC, supporting the prevailing view that foot placement significantly influences frontal plane CoM motion. Our approach enhances this understanding by quantifying its relative contribution to final CoM transfer magnitude asymmetry. We found that the projected CoM transfer magnitude asymmetry at IC accounts for approximately 53.9% of the final CoM transfer magnitude asymmetry. Given that the influence of foot placement is about ten times greater than that of CoM velocity to projected CoM transfer magnitude asymmetry at IC, the contribution of foot placement to final CoM transfer magnitude asymmetry can be calculated as $53.9\%\times \frac{10}{10+1}\approx 49.0\%$. Additionally, the disparity in the scale of influence between foot placement and CoM velocity primarily stems from the difference in their respective coefficients in (1). This indicates that while a faster CoM velocity during CoM transfer towards the paretic side could potentially mitigate the effects of wide foot placement, its impact is approximately ten times smaller. Quantitatively, to counteract a 1 cm increase in foot placement, a 0.1 m/s increase in CoM velocity is required. Our data showed that CoM velocity at IC averaged approximately 0.15 m/s. Consequently, an increment of 0.1 m/s would represent a 67% increase, which realistically might be very challenging for individuals post-stroke to voluntarily achieve.

### B. Temporal Evolution of CoM Transfer Asymmetry (H1) and Kinetically Derived Work Input (H3)

Contrary to our hypothesis that compensation for projected CoM transfer magnitude asymmetry established at IC would occur during the double support phase, we found that the projected CoM transfer magnitude asymmetry was instead augmented during this phase. This was primarily due to an asymmetry in work input from the trailing limb. We found that the work input from the trailing limb could explain over 50% of the variance in changes in projected CoM transfer magnitude during the double support phase, and the non-paretic trailing limb generated less work, power, and force compared to the paretic limb. This reduced force generation from the non-paretic side is counterintuitive, given its typically greater force generation capacity compared to the paretic limb [43]. However, a recent study similarly found that the non-paretic limb generated less lateral ground reaction force (GRF) than the paretic limb during walking [44]. One explanation for this observation could be a conservative strategy to reduce CoM transfer magnitude and decrease the risk of lateral instability during the paretic steps. Studies in foot placement indicate that individuals post-stroke often place their feet wider without actively controlling it based on the CoM state as exhibited in healthy controls. This strategy may help prevent lateral falls and is robust against disrupted proprioceptive sensory feedback, but the prioritization of stability often comes at the cost of efficiency [12], [24]. In line with this strategy, our results suggest that the reduced mediolateral force generation at the non-paretic limb is likely a conservative approach to prevent falling in addition to a wide paretic foot placement.

Notably, all participants in this study walked at a slower speed on the treadmill (0.33 ± 0.13 m/s) compared to their overground walking speed measured during the 10-Meter Walk Test (0.71 ± 0.29 m/s). This reduced speed is likely attributable to participants walking without handrails during our protocol, whereas during the 10MWT, participants were permitted to use their typical assistive devices as typically done in clinical settings (walkers, canes, or trekking poles; Table I). This discrepancy suggests an alternative explanation for the reduced non-paretic trailing limb work observed in our study. Participants who habitually ambulate with a cane in their non-paretic hand may have developed a compensatory pattern of reduced lower-limb effort on that side. When transitioning to hands-free treadmill walking, they may have continued to offload their non-paretic lower limb. Future studies investigating whether an extended adaptation period to hands-free walking results in increased non-paretic trailing limb work and altered CoM transfer dynamics are warranted.

Although we focused on trailing limb work input in the present study, it is worth noting that the leading limb could also contribute to changes in projected CoM transfer magnitude during the double support phase. However, we derived a simple equation for CoM transfer magnitude projection under the assumptions of the LIPM. In this framework, the leading limb is approximated as a passive structure, with its motion constrained to maintain a constant CoM height. Under this assumption, the energy contribution from the leading limb primarily involves transferring kinetic energy to potential energy to decelerate the pendulum, without introducing additional mechanical energy into the system. Disentangling active energy contributions from the passive dynamics of the leading limb would require a more complex model, such as incorporating an independently actuated telescoping leg or adding a flywheel at the CoM to model trunk control via the leading hip joint. However, these additions would compromise the simplicity of the current framework, which we leveraged to capture the temporal characteristics of CoM transfer asymmetry. Our findings highlight the critical role of changes in the CoM state during the double support phase in shaping CoM transfer magnitude and variability asymmetry. While we demonstrated that energy contributions from the trailing limb account for a substantial portion of these changes, future studies are warranted to build on these insights by investigating the combined contributions of both limbs during the double support phase using more advanced biomechanical models.

### C. Step-by-Step Control of Work Input during Double Support Phase (H4)

We observed a linear association between the projected CoM transfer magnitude at IC and its adjustment in the double support phase for both sides. This indicates that the CoM transfer magnitude is consistently re-calibrated after IC, likely with enhanced work input from the trailing limb when projected transfer magnitudes are lower. Furthermore, we found that this association was weaker during the paretic steps, suggesting a disrupted active control mechanism. Our analysis of variability highlighted that although continuous adjustment of the CoM state after IC could attenuate variability, a diminished control mechanism during the paretic steps likely led to the persistent higher variability from IC to transfer completion compared to non-paretic steps. Given the expected less compromised motor output accuracy in the non-paretic lower limb [45], the observed failure to reduce variability with active control likely stems from disrupted somatosensory feedback in the paretic stance limb [46]. This sensory impairment may lead to a more conservative compensatory strategy to manage risk of frontal plane instability, characterized by wider foot placement and reduced trailing limb work input in paretic steps. These insights could inform three potential directions for improving walking performance in individuals post-stroke. Firstly, the disrupted ongoing adjustment of the projected CoM transfer magnitude could be compensated for by wearable assistive devices that monitor the projected CoM transfer magnitude during each step and adjust it with trailing limb work input. A similar prosthetic controller design has been demonstrated to improve balance in people with amputation [27]. Secondly, enhancing proprioceptive feedback through techniques such as vibratory sensory augmentation [47], [48] or biofeedback [49], [50] could facilitate tighter regulation of CoM transfer magnitude. Lastly, while adjusting foot placement is a viable strategy to control mediolateral CoM motion, it is noteworthy that our findings emphasize the need to consider ongoing adjustments during the double support phase as an adaptation strategy for individuals post-stroke to counteract the modulating effect of foot placement.

### D. Limitation

There are several limitations to the present study. Firstly, the analysis relied on the assumptions of the LIPM, such as a constant CoM height. However, we did not observe a significant CoM height changes throughout the transfer phase in our participants (0.72 ± 0.26% and 1.27 ± 0.41% body height for transfers towards the paretic and non-paretic sides, respectively). Additionally, the predictive accuracy of the model during the early single stance phase suggests that its foundational assumptions were not significantly violated. Secondly, it is worth noting that the trailing limb work input accounted for approximately 50% of the variance in changes to the projected CoM transfer magnitude, and the relationship did not exhibit a one-to-one correspondence. This implies that other dynamics, such as changes in angular momentum or active energy contributions from the leading limb, could also influence the CoM state during the double support phase. However, to maintain analytical consistency within the LIPM framework, these factors were not explored in this study. Thirdly, our analyses were conducted under conditions that did not involve the use of a handrail, which likely led to slower treadmill walking speeds compared to overground speeds, where hand-held assistive devices were permitted. While this approach may not fully replicate real-world ambulation, it was chosen to establish foundational knowledge toward achieving the ultimate goal of restoring ambulation without reliance on handrails or canes. However, the impact of handrail or cane use and walking speed on CoM transfer dynamics remains to be determined. Lastly, we acknowledge that our study’s relatively small sample size limits the generalizability of our findings. This limitation arose in part from our focus on walking without handrail support to better capture the inverted-pendulum-like dynamics of typical gait, which posed recruitment challenges. Nevertheless, our dataset reflects significant functional heterogeneity, with Berg Balance Scale scores ranging from 17 to 51 and 10-meter walk test speeds from 0.16 to 1.02 m/s. Despite its size, this dataset offers valuable preliminary insights into unassisted post-stroke gait that warrants further investigation with larger cohorts to validate and expand on our findings.

## V. Conclusion

In conclusion, our findings partially support our central hypothesis. Specifically, we found that over half of the asymmetry in CoM transfer magnitude was established at initial contact, confirming that diminished CoM transfer originates early in the paretic steps. However, contrary to our central hypothesis, the non-paretic trailing limb did not compensate for this asymmetry through positive work input or step-to-step variability attenuation. Our study has underscored that adjustments during the double support phase are crucial determinants of asymmetries in frontal plane CoM transfer magnitude and variability in individuals post-stroke. Additionally, the LIPM-based approach we introduced enables prediction of CoM transfer magnitude within each step, which could have broader applications in rehabilitation and assistive device design.

## Figures and Tables

**TABLE I:** Participant Demographic Information

Subject ID	CWS (m/s)	Age (years)	Time Since Last Stroke (years)	Dominant Side	Paretic Side	Sex	Assistive devices used during treadmill walking	10MWT (m/s)	Berg Balance Score	Additional devices used during clinical test
s01	0.4	56.5	7.6	Right	Left	M	Bioness FES at left shank	1.02	39	Trekking pole
s02	0.25	34.7	7.9	Right	Left	F	-	0.9	36	-
s03	0.5	75.8	0.7	Right	Right	M	AFO at right ankle	0.99	39	Trekking pole
s04	0.45	68.4	2.2	Right	Right	F	-	0.91	51	-
s05	0.25	49.1	2.7	Right	Left	F	AFO at left ankle	0.46	33	Single point cane
s06	0.5	51.3	9.4	Left	Left	M	-	0.65	29	-
s07	0.25	60.0	2.0	Right	Right	F	-	0.93	45	-
s08	0.2	59.6	7.2	Right	Left	M	AFO at left ankle	0.3	39	Single point cane
s09	0.25	73.0	2.6	Right	Left	M	-	0.96	29	Rolling walker
s10	0.3	58.9	6.5	Right	Left	F	Bioness FES at left shank	0.59	45	Trekking pole
s11	0.5	45.4	0.7	Right	Left	M	-	0.92	51	-
s12	0.1	54.6	1.7	Left	Left	F	AFO at left ankle	0.16	17	Quad cane
s14	0.35	76.9	1.1	Right	Right	M	AFO at right ankle	0.43	49	-
Mean	0.33	58.8	4.0	-	-	-	-	0.71	38.6	-
SD	0.13	12.4	3.2	-	-	-	-	0.29	9.9	-

Participants with larger CoM transfer magnitude during paretic versus non-paretic step thus excluded from the analysis in Result sections B-E are highlighted in blue. *AFO: Ankle-foot-orthosis. FES: Functional electrical stimulation. CWS: Comfortable treadmill walking speed. 10MWT: Ten-meter overground walk test. SD: Standard deviation.*

**TABLE II:** List of Initial Hypotheses and Whether They Are Supported by Our Results

Hypothesis	Statistical Analyses and Results Interpretation
**H1:** The projected CoM transfer magnitude asymmetry would be the highest at IC, decrease at CFO, and remain consistent from CFO to transfer completion.	**Statistical Test:** One-way repeated measures ANOVA showed a significant main effect of gait event (IC, CFO, CoM transfer completion) on CoM transfer asymmetry, followed by pairwise post-hoc comparisons. **Supporting:** CoM transfer asymmetry did not significantly differ between CFO and transfer completion (*p* = 0.09; pairwise post-hoc comparison), supporting the hypothesis that asymmetry remains consistent from CFO to transfer completion. **Not supporting:** CoM transfer asymmetry increased, not decreased, from IC to CFO (*p* < 0.01; pairwise post-hoc comparison), contradicting the hypothesis. **Additional Findings:** Projected CoM transfer magnitude asymmetry at IC accounted for 53.9% of the final asymmetry, while changes from IC to CFO contributed 38.1%.
**H2:** Individuals post-stroke would exhibit wider foot placement and decreased lateral CoM velocity during paretic compared to non-paretic IC.	**Statistical Test:** Two linear mixed-effects models were used: Model 1: FootplacementatIC∼Transferdirection+(1∣SubjectID) Model 2: LateralCoMvelocityatIC∼Transferdirection+(1∣SubjectID) **Supporting:** A significant fixed effect of transfer direction (*p* < 0.01) showed that individuals post-stroke had wider foot placement at paretic versus non-paretic IC. **Not Supporting:** A significant fixed effect of transfer direction (*p* < 0.01) showed that individuals post-stroke had faster, rather than slower, lateral CoM velocity at paretic versus non-paretic IC. **Additional Findings:** Foot placement had approximately 10 times greater influence on projected CoM transfer magnitude than CoM velocity at IC, primarily due to the coefficients in the LIPM-based equation.
**H3:** Changes in projected CoM transfer magnitude during DS would be associated with trailing limb work input, and the non-paretic trading limb would exhibit higher work input due to greater force generation compared to the paretic trailing limb.	**Statistical Test:** Simple linear regression quantified the variance in changes in projected CoM transfer magnitude explained by kinetically derived trailing limb work input during DS. Four linear mixed-effects models (DependentVariableTransferdirection+(1∣SubjectID)) tested the significance of transfer direction, each with a different dependent variable: trailing limb work input, average power, mediolateral ground reaction force, and CoM velocity during double support. **Supporting:** Trailing limb work input explained over 50% of the variance in changes in projected CoM transfer magnitude during DS. **Not supporting:** Significant fixed effects (*ps* < 0.01) of transfer direction in each model indicated that the non-paretic trailing limb generated less rather than greater work, power, and force compared to the paretic limb.
**H4.1:** A stronger relationship between the projected CoM transfer magnitude at IC and its changes during DS in paretic versus non-paretic steps.	**Statistical Test:** Significance of transfer direction tested with a linear mixed-effects model: CorrelationcoefficientbetweentheprojectedCoMtransfermagnitudeatICanditschangesduringDS∼Transferdirection+(1∣SubjectID). **Not supporting:** A significant fixed effect (*p* < 0.01) of transfer direction showed that the correlation coefficient was lower rather than higher for paretic steps compared to non-paretic steps.
**H4.2:** A reduction in CoM transfer magnitude variability from IC to CoM transfer completion in both paretic and non-paretic steps, with a greater reduction in paretic steps.	**Statistical Test:** A linear mixed-effects model: CoMTransferVariability∼TransferDirection(pareticvs.non-paretic)+TimePoint(ICvs.transfercompletion)+(TransferDirection×TimePoint)+(1∣SubjectID) revealed a significant main effect of time point (*p* < 0.05) and its interaction with transfer direction (*p* < 0.05), followed by planned post-hoc comparisons of CoM transfer variability between transfer directions at each time point and across time points within each transfer direction. **Supporting:** Planned post-hoc comparisons across time points within each transfer direction showed variability was reduced (*p* < 0.01) from IC to transfer completion in non-paretic steps. **Not supporting:** Post-hoc comparisons across time points within each transfer direction showed no significant change in variability from IC to transfer completion in paretic steps (*p* = 0.99). Post-hoc comparisons between transfer directions at each time point showed that variability was greater when transferring towards the paretic versus non-paretic side at transfer completion ((*p* < 0.05), but no difference was found at IC ((*p* = 0.75).

IC: Initial contact. CFO: Contralateral foot off. DS: Double support.
